# Crystal structure of APOBEC3A bound to single-stranded DNA reveals structural basis for cytidine deamination and specificity

**DOI:** 10.1038/ncomms15024

**Published:** 2017-04-28

**Authors:** Takahide Kouno, Tania V. Silvas, Brendan J. Hilbert, Shivender M. D. Shandilya, Markus F. Bohn, Brian A. Kelch, William E. Royer, Mohan Somasundaran, Nese Kurt Yilmaz, Hiroshi Matsuo, Celia A. Schiffer

**Affiliations:** 1 Department of Biochemistry and Molecular Pharmacology, University of Massachusetts Medical School, Worcester, Massachusetts 01655, USA; 2 Program in Molecular Medicine, University of Massachusetts Medical School, Worcester, Massachusetts 01655, USA; 3 Basic Research Laboratory, Leidos Biomedical Research, Inc., Frederick National Laboratory, Frederick, Maryland 21702, USA; 4 Department of Biochemistry, Molecular Biology and Biophysics, Institute for Molecular Virology, University of Minnesota, Minneapolis, Minnesota 55455, USA

## Abstract

Nucleic acid editing enzymes are essential components of the immune system that lethally mutate viral pathogens and somatically mutate immunoglobulins, and contribute to the diversification and lethality of cancers. Among these enzymes are the seven human APOBEC3 deoxycytidine deaminases, each with unique target sequence specificity and subcellular localization. While the enzymology and biological consequences have been extensively studied, the mechanism by which APOBEC3s recognize and edit DNA remains elusive. Here we present the crystal structure of a complex of a cytidine deaminase with ssDNA bound in the active site at 2.2 Å. This structure not only visualizes the active site poised for catalysis of APOBEC3A, but pinpoints the residues that confer specificity towards CC/TC motifs. The APOBEC3A–ssDNA complex defines the 5′–3′ directionality and subtle conformational changes that clench the ssDNA within the binding groove, revealing the architecture and mechanism of ssDNA recognition that is likely conserved among all polynucleotide deaminases, thereby opening the door for the design of mechanistic-based therapeutics.

## Abstract (web summary)

Cytidine deaminases are evolutionarily conserved enzymes that edit genomes by deaminating cytidine to uridine. Here the authors present the crystal structure of APOBEC3A with a single-stranded DNA substrate bound in the active site to shed light on the mechanism and specificity of substrate recognition.

Apolipoprotein B messenger RNA-editing enzyme, catalytic polypeptide-like (APOBEC3) proteins are single-stranded DNA (ssDNA) deoxycytidine deaminases that are among some of the fastest evolving proteins in the human genome^1^. APOBEC3s catalyse a cytidine (C) to uridine (U) zinc-dependent deamination reaction^2^,^3^,^4^,^5^. The seven APOBEC3 enzymes are clustered on chromosome 22 (ref. 6). Although each APOBEC3 has a single catalytic active site, the human genome includes three single-domain (APOBEC3A, C and H) and four double-domain (APOBEC3B, D, F and D) enzymes. The double-domain enzymes consist of a catalytically active C-terminal domain (CTD) and an inactive pseudo-catalytic N-terminal domain (NTD) that can bind but not edit nucleic acids. Four of the seven APOBEC enzymes (APOBEC3D, APOBEC3F, APOBEC3G and APOBEC3H) have been implicated as HIV-1 host restriction factors^7^,^8^,^9^,^10^,^11^,^12^,^13^. The APOBEC3 enzymes act on ssDNA to introduce C-to-U modifications that create G-to-A point mutations on the paired strand as the U is read as T during replication. Such mutations in ssDNA can lead to double-strand breaks that may result in genomic DNA damage that have been observed in cancer^14^,^15^,^16^,^17^,^18^,^19^,^20^.

In the last decade, our laboratories^21^,^22^,^23^,^24^,^25^,^26^,^27^ along with others^28^,^29^,^30^,^31^,^32^,^33^,^34^,^35^,^36^,^37^,^38^,^39^,^40^,^41^,^42^ have solved crystal and nuclear magnetic resonance (NMR) structures of single domains of human APOBEC3s (Supplementary Fig. 1). These proteins share the same overall fold^43^, deaminate cytosines in ssDNA, but vary in their substrate specificity, processivity, catalytic rate and ability to restrict HIV-1. All APOBEC3 domains contain a H_A_E_*x*28_C_*x*2-4_C zinc binding motif. The carboxylate group of the catalytic glutamic acid stabilizes the transition state and proton transfer during catalysis where a water coordinated by the catalytic zinc is the sole source of proton for the amino group and N3 atom of cytosine^2^,^44^,^45^. The specificity of different APOBECs has been elucidated by the determination of preferred mutagenic hotspot sequences, 5′-CC/TC-3′ for APOBEC3A (studied here)^46^, 5′-TC-3′ for APOBEC3F and 5′-CC-3′ for APOBEC3G^10^,^47^,^48^. APOBEC3G deaminates hotspots closer to 5′-end more efficiently than to 3′-end of ssDNA^28^,^30^,^32^,^49^, but the underlying mechanism for this preference is not known. Several alternative ssDNA-binding models for APOBEC3G-CTD and APOBEC3A have been proposed^21^,^29^,^35^,^36^. Most recently, the crystal structure of the inactive pseuodo-catalytic rhesus macaque APOBEC3G-NTD (rA3G-NTD) (Supplementary Fig. 1b) in complex with poly-dT ssDNA has been reported^42^. However, only one complete deoxythymidine (dT) was resolved in this structure bound in a shallow cleft far from the pseudo-catalytic zinc-binding motif. This complex did not reveal how substrate (dC) or product (dU) may be accommodated for deamination reaction. The details of ssDNA-binding and -editing mechanisms, and molecular basis underlying substrate nucleotide sequence specificities of APOBEC3 enzymes still remain elusive.

APOBEC3A (A3A) is a single-domain enzyme with the highest catalytic activity among the human APOBEC3 proteins^50^. While the DNA-editing activity inhibiting the replication of retroelements is beneficial for genome stability, increased expression or defective regulation of A3A could lead to mutagenesis of human genome and contribute to carcinogenesis^51^. The structure of A3A was initially determined by NMR^35^ and some preference for DNA over RNA was suggested by chemical shift perturbation data^36^. However, mutations of residues predicted to be involved in DNA targeting had variable effects on deamination activity, and the detailed mechanism by which A3A binds DNA substrate is still elusive^35^,^36^.

In this study, we determined the crystal structure of a ssDNA:deaminase complex, or a polynucleotide substrate bound at the active site of a catalytic domain APOBEC3 protein. Previously, we solved the crystal structure of the unliganded inactived A3A (ref. 26) and determined potent binding affinity to substrate ssDNA of ∼60 nM, whereas the product exhibited an order of magnitude lower affinity. Here the crystal structure to 2.2 Å of this variant of A3A in complex with substrate DNA oligonucleotide containing a single 5′-TC-3′ deamination target sequence in a polyT background is presented. The central nucleotides comprising the 5′-TCT-3′ motif is well ordered and bound at the active site, revealing the intermolecular interactions defining specificity for the bases at each of these three positions. The target deoxycytidine (dC_0_), is bound in a reaction-competent coordination at the active site. This A3A–ssDNA structure elucidates the molecular basis of nucleotide preferences in the substrate motif and provides key insights into the overall molecular mechanisms of DNA editing by cytidine deaminases.

## Results

### A3A–ssDNA co-crystal structure

**Figure 1:**
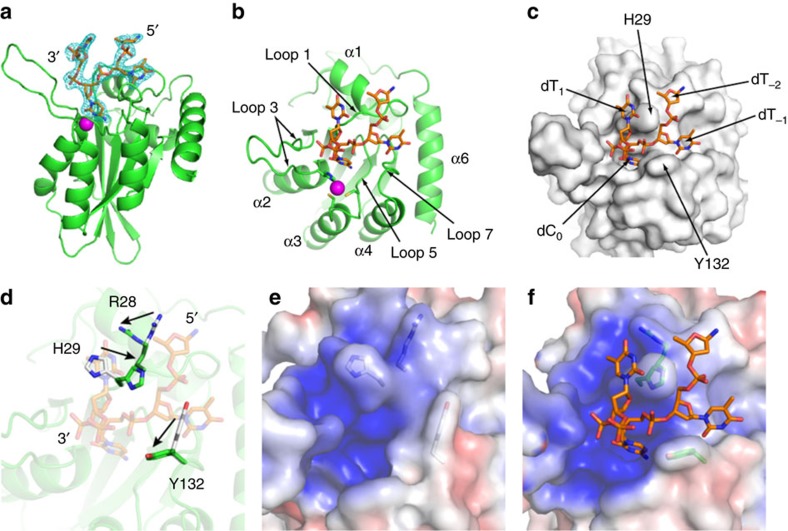
Crystal structure of A3A in complex with substrate DNA. (**a**) A3A structure with a 2*Fo*−*Fc* electron density map contoured at 1*σ*. The protein is presented as a green-coloured ribbon diagram and the bound DNA is in stick representation (carbons and phosphates, orange; nitrogens, blue; oxygens, red). A zinc ion at the active centre is depicted as a magenta-coloured sphere. The side chains of zinc-coordinating residues H70, C101 and C106 are shown as sticks (carbons, green; nitrogens, blue; oxygen, red; sulfurs, yellow). DNA binding at the active site of A3A is presented in (**b**) ribbon and (**c**) surface representation. (**d**) Conformational changes of residues R28, H29 and Y132 upon DNA binding are indicated by arrows, with side chains in stick representation (white and green-coloured carbon for the apo (PDB code 4XXO)^26^ and DNA-bound forms, respectively). Surface electrostatic potentials of (**e**) apo and (**f**) DNA-bound A3A are coloured red to blue for negative and positive charges, respectively, using a scale of −5 to +5 kT *e*^−1^.

A3A (E72A/C171A)^26^ was used for co-crystallization with ssDNA. E72A inactivates the enzyme permitting the formation of stable complexes and C171A increases solubility. The crystal structure of A3A (E72A/C171A) in complex with ssDNA was determined by molecular replacement at 2.2 Å resolution (Fig. 1a–c; Supplementary Fig. 2). A 15-mer DNA oligonucleotide that binds A3A with ∼60 nM affinity^26^ with a target deoxycytidine (5′-TTTTTTTCTTTTTTT-3′) was co-crystallized with A3A. The final refinement of the structure resulted in R-factor/R-free of 0.177/0.225, respectively (Table 1).

There was a single A3A–ssDNA complex in the asymmetric unit and crystal contacts with symmetry-related complexes did not correspond to the zinc-coordinated dimer interface we observed for the apo A3A crystal structure^26^. The apo A3A structure included an excess of zinc (50 μM ZnCl) in the crystallization condition, while the A3A–ssDNA complex lacked added zinc, which may have destablized the dimer within this crystal form. The cooperativity upon DNA binding we observed in solution and interrogated with site-directed mutagenesis^26^ implicates A3A capable of binding ssDNA in the dimeric form at least transiently. Nevertheless, cooperativity does not seem to be essential as the monomeric form of A3A, with a mutation at H56A, binds substrate DNA with similar affinity^26^. Most likely both monomer and dimer forms of A3A play a role in recognizing substrates in solution.

The target deoxycytidine (dC_0_) and flanking deoxythymidines (dT_−1_ and dT_1_), as well as one additional deoxyribose at 5′-end and one phosphate at 3′-end, were well ordered in the electron density (5′-sugar-dT_−1_-dC_0_-dT_1_-phosphate-3′; Fig. 1a). Of the nearly 1,280 Å^2^ of surface area on the resolved DNA, ∼620 Å^2^ is buried in the interface with A3A. The central cytidine (dC_0_) and the preceding thymidine (dT_−1_) are accommodated in a deep groove formed by Loops 1,3,5 and 7 of A3A (Fig. 1b; Supplementary Fig. 1a). The bound DNA adopts an irregular conformation to encircle the side chain of H29 (Fig. 1c). Compared to apo A3A, there are conformational changes in the rotamers of the side chains of R28 and H29 in Loop 1, and Y132 in Loop 7, accompanied by more subtle reorganization of N57–A72 in loop 3 (Fig. 1d; Supplementary Figs 1 and 3). The rest of the enzyme including the active site remains essentially unchanged. The groove significantly differs from any of the previously suggested models for how ssDNA binds to A3s (refs 21, 29, 35, 36) including the recent structure of the pseuodo-catalytic A3G-NTD in complex with poly-dT ssDNA^42^. This conformational change allows the groove to sequester the ssDNA by forming a more complementary molecular surface, both in terms of van der Waals packing and electrostatic (electropositive) nature of the groove (Fig. 1e,f).

### Recognition of the targeted cytidine

**Figure 2:**
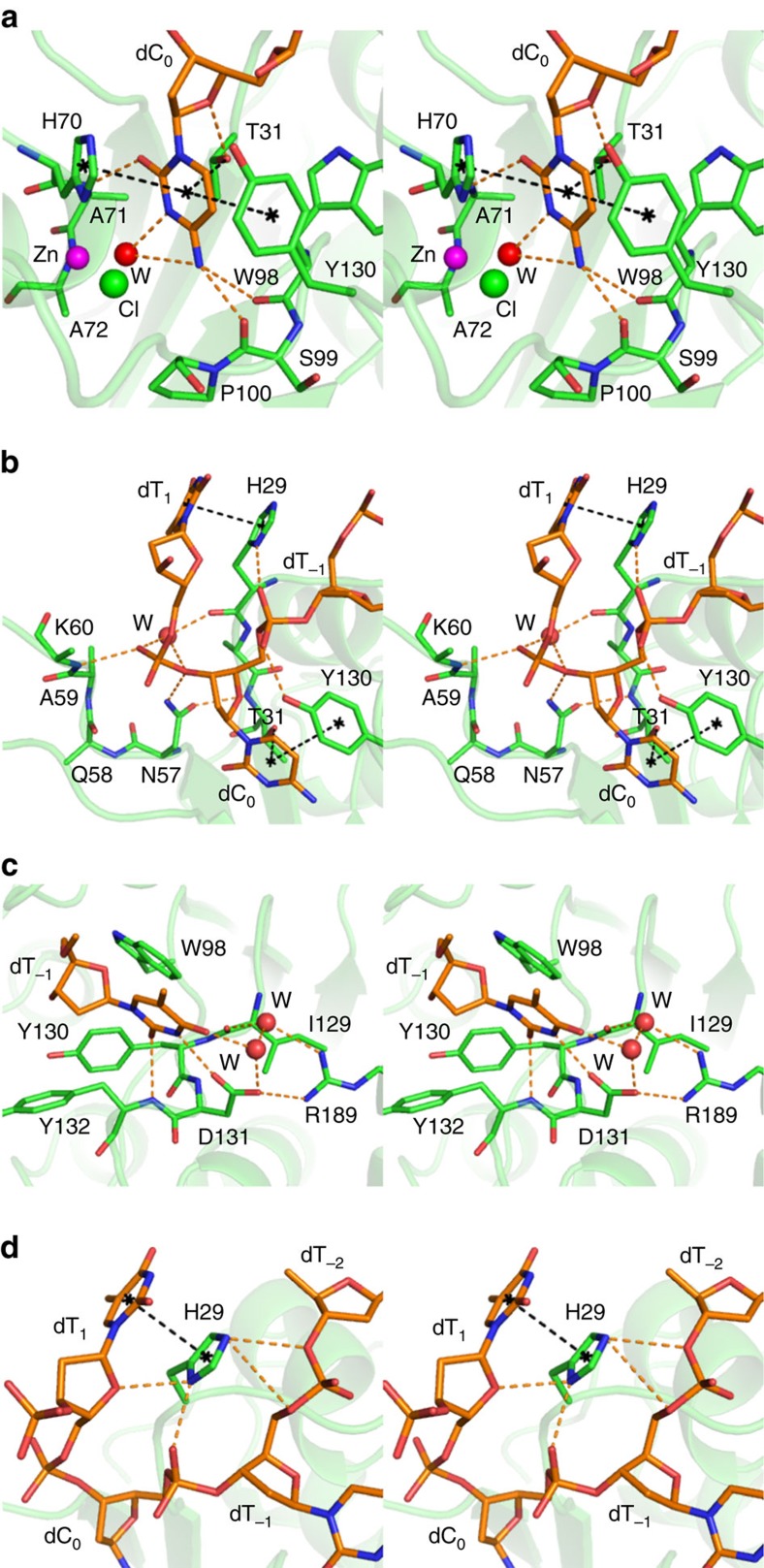
A3A–ssDNA atomic interactions. Stereo-view of the interactions between A3A and (**a**) the target nucleotide base (dC_0_), (**b**) the DNA backbone flanking dC_0_ (**c**) nucleotide at −1 position (dT_−1_). (**d**) Interactions between H29 side chain and the substrate DNA. Side chains of A3A residues (carbons green) and the DNA (carbons and phosphates orange) are in stick representation, with other atoms coloured as in Fig. 1b. A zinc ion (Zn) at the active centre, the zinc-liganded chlorine (Cl) and water molecule (W) are indicated by spheres coloured magenta, green and red, respectively. Estimated hydrogen bonds and π–orbital interactions are depicted by dashed lines coloured orange and black, respectively.

The deoxycytidine (dC_0_), which is the target of deamination reaction, is well coordinated and buried within the active site of A3A. The cytidine ring is located directly over the hydroxyl group of the T31 side chain, which likely hydrogen bonds to the π-orbital cloud of the base ring and simultaneously coordinates O4 atom of the deoxyribose (Fig. 2a). Residue Y130 contributes to the dC_0_ positioning by forming a T-shaped π–π interaction with the pyrimidine ring. The hydroxyl group of Y130 further forms a hydrogen bond with 5′-phosphate of dC_0_ (Fig. 2b). The H70 side chain is positioned over the N1 atom of dC_0_, capable of potentially forming a π–π stacking (Fig. 2a). The backbone NH of A71 hydrogen bonds to O2 of dC_0_. In addition, the carbonyl oxygen atoms of W98 and S99 form a bifurcated hydrogen bond to NH_2_ of the cytosine, which appears to both support the dC_0_ positioning and dictate the specificity for cytosine over thymine.

**Figure 3:**
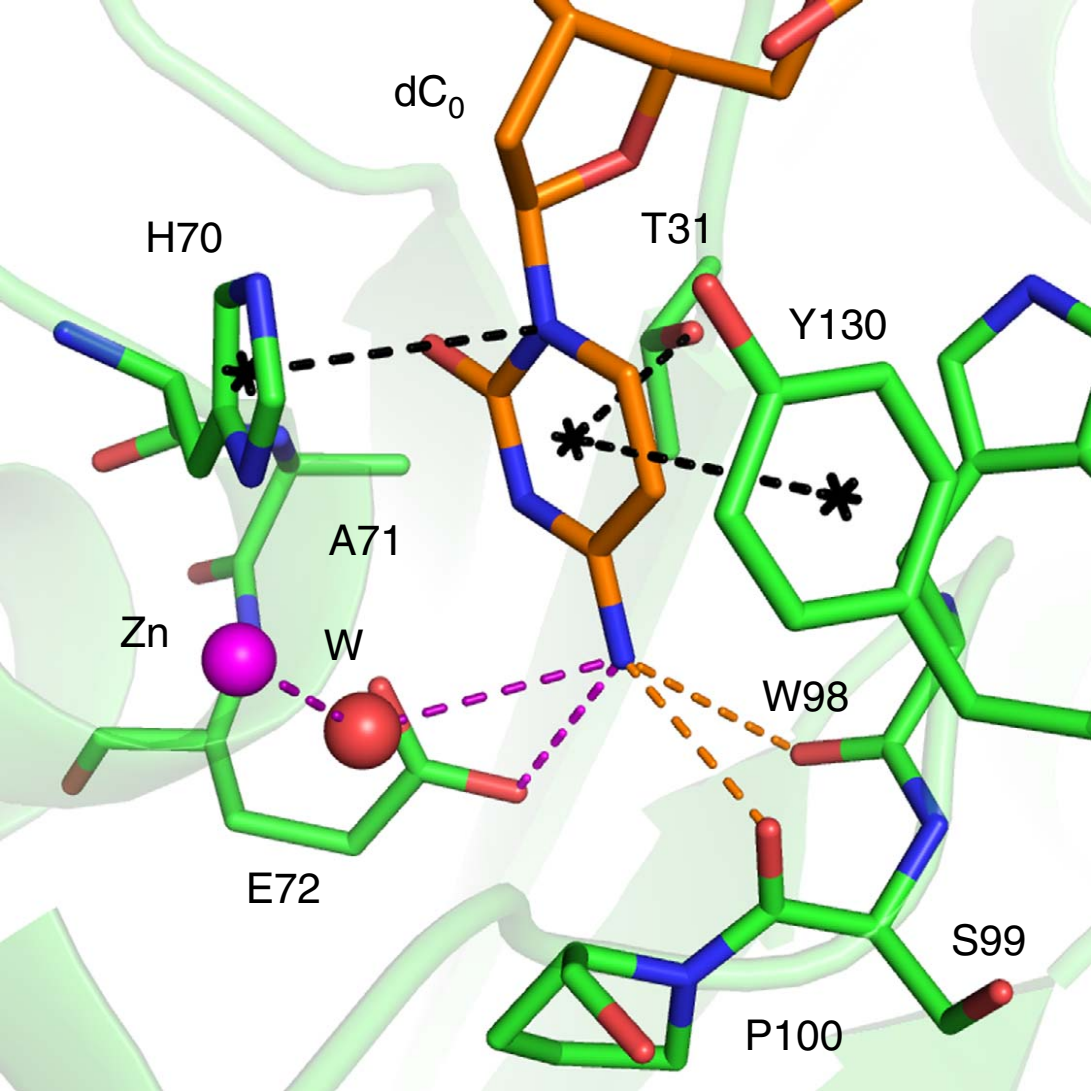
Structural model of the A3A catalytically active site. The target nucleotide base (dC_0_) bound at the A3A active site where the catalytic E72 side chain was modelled in instead of the alanine at this position in the crystal structure. Zinc, the coordinated water (W), carbonyl oxygen of dC_0_ and carboxyl oxygen of E72 side chain were connected by dashed lines in magenta.

As expected for a catalytic A3 domain, electron density that fits a zinc ion was observed coordinating H70, C101, C106 as well as additional density that fits a Cl^−^ ion, with both assignments confirmed by anomalous difference calculations. To prevent catalysis, our A3A construct was inactivated by an E72A mutation, which left the geometry of the active site intact (Fig. 2a). Instead of the E72 side chain, we observe electron density that fits a water molecule. Molecular modelling of E72 into this space shows the side chain would be positioned just proximal to the deamination target, C4-NH_2_ moiety, of the cytosine (Fig. 3) and poised for deamination reaction^2^. After catalysis and subsequent release of NH_3_, this coordination, along with the interactions with W98 and S99, would be unfavourable for the product uridine. Overall, multiple interactions of the substrate cytosine with A3A active site residues ensure the specific recognition and geometry required for the deamination reaction and product release.

### Specificity for pyrimidines at −1 position

The deoxythymidine at the 5′-side of the target (the −1 position; dT_−1_) has extensive van der Waals contacts with three residues from Loop 7 (Y130, D131 and Y132) and W98 in Loop 5 (Fig. 2c). The Watson–Crick edge of the thymine base faces these Loop 7 residues, and makes three hydrogen bonds: O2 atom with Y132 backbone amide, N3 with the D131 side chain carboxylate and O4 with a water molecule. In addition, the D131 side chain has a salt bridge to the R189 side chain in helix 6, which stabilizes the overall hydrogen bonding configuration of Loop 7 to the thymine base. This coordination appears critical as residue 189 is conserved as a basic residue (Arg/Lys) only in catalytically active A3 domains (Supplementary Fig. 1; Supplementary Table 1). At the −1 position, deoxcytidine could form similar, but slightly rearranged, interactions as the N3 atom lacks the proton to hydrogen bond with D131. Indeed, although A3A has dual specificity for 5′-TC-3′ and 5′-CC-3′ (ref. 40), there is a preference for thymidine at the −1 position. However, Loop 7 of A3A, in particular residues Y130 and D131, would likely preclude a larger purine base from fitting in this position, thus defining the T/C specificity of A3A.

### The conserved N57 is central to the active site geometry

N57 of A3A is completely conserved among the catalytically active APOBEC protein domains, while inactive pseudo-catalytic A3 domains have a conserved glycine (Supplementary Fig. 1; Supplementary Table 1), and widely conserved among other cytidine/cytosine deaminases from *Escherichia coli* through *Homo sapiens*^52^. The structure explains this strong conservation, as N57 of A3A is central in recognizing ssDNA with three key distinct interactions: The side chain of N57 determines the 5′–3′ directionality of ssDNA binding by forming a hydrogen bond to O3′ atom of dC_0_, which helps stabilize the geometry of the DNA backbone and the sugar in a C2′-endo conformation (Fig. 2b; Supplementary Fig. 4a) and induces a backbone deformation due to steric hindrance with O5′ of the target dC_0_. The N57 side chain forms a hydrogen bond with the backbone NH of T31, positioning the T31 side chain to hydrogen bond to the π-orbital cloud of the dC_0_ base ring, thus ensuring the geometry of the target nucleotide within the active site (Fig. 2a; Supplementary Fig. 4a). Finally, the N57 side chain packs against both the deoxyribose ring of dC_0_, stabilizing the orientation of sugar plane, and H70, which coordinates zinc. Although RNA deaminase activity has been reported for A3A^53^,^54^, if the sugar was a ribose a steric clash between the 2′-OH and H70 would occur, therefore requiring a conformational rearrangement for RNA modification. Thus, these three pivotal interactions of N57 organize the enzyme substrate complex to be poised for catalytic turnover.

The three central interactions mediated by N57 are strictly conserved in the active site geometry of other cytidine deaminases^55^,^56^,^57^, where the asparagine side chain (1) hydrogen bonds to substrate backbone, (2) packs to maintain the sugar orientation and (3) packs against the zinc-coordinating residue side chain (Supplementary Fig. 4b–d). The RNA cytidine deaminases replace the zinc coordinating histidine with a relatively small amino acid, cysteine, which permits a ribose ring to fit (Supplementary Fig. 4c,d). This structure explains why although not located directly at the active site, even conservative N57Q or N57D mutations severely disrupt deaminase activity^29^,^52^,^58^, thus our A3A–ssDNA structure reveals the conservation of N57 to be critical for proper orientation of the substrate within the active sites of cytindine deaminases.

### H29 coordinates the ssDNA binding in the active site

H29 is the other lynchpin in ssDNA binding to A3A. H29 of A3A corresponding to H216 in the catalytic domain of A3G (A3G-CTD), which when mutated to alanine abolishes activity^21^. Maximal catalytic activity occurs at pH 5.5 for both A3G-CTD^59^ and A3A^51^, implying that the histidine is protonated. Interestingly, this His is not completely conserved in other A3s, where this position is sometimes an arginine or asparagine. The H216R mutation in A3G and H29R in A3A resulted in reduced but still significant catalytic activity^41^,^59^. In the apo A3A crystal structure^26^, H29 is involved in crystal contacts and rotated away from the active site (Fig. 1d). In the NMR structure of A3A the H29, side chain is solvent exposed and the rotamer is not defined in solution (PDB code 2M65)^35^. Thus, upon ssDNA binding, the side chain of H29 selects a rotamer to interact extensively with the substrate, latching the active site to permit catalysis. Once catalysis occurs, H29 needs to rotate out of this position to release the deaminated product. H29 forms hydrogen bonds to the backbone phosphates of dT_−1_, dC_0_, and dT_1_, and the deoxyribose of dT_1_ (Figs 1d–f and 2d). The side chain of H29 is crucial in dT_1_ recognition, with the imidazole ring positioned to form π–π interactions with the pyrimidine ring of dT_1_. This relatively non-specific stacking interaction explains the apparent lack of specificity at the +1 position. Thus, our structure reveals the unique role of H29 in positioning the substrate ssDNA with a series of coordinated hydrogen bonds and stacking interactions, essentially latching the ssDNA and the target dC_0_ within the active site.

### A3A and rA3G-NTD differ in DNA binding

**Figure 4:**
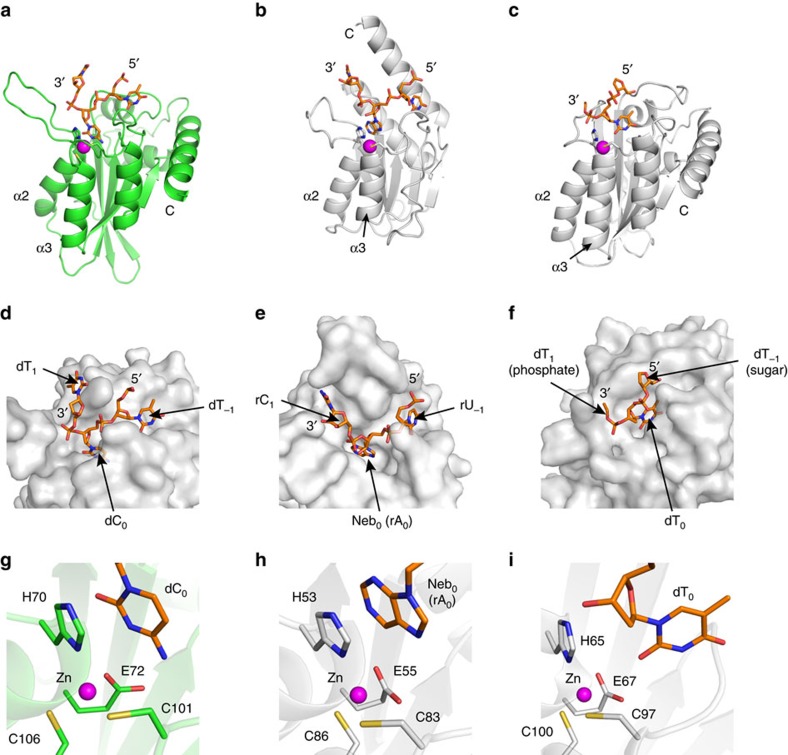
Structure and substrate-binding similarity between A3A and RNA deaminase TadA. (**a**) A3A structure (green ribbon) bound to substrate DNA (orange sticks, as in Fig. 1b). Three DNA nucleotides (dT_−1_, dC_0_ and dT_1_) are displayed. A zinc ion at the active centre is coordinated by H70 (helix α2), C101 and C106 (helix α3). (**b**) TadA structure^60^ (PDB code 2B3J) (grey ribbon) bound to substrate RNA (orange sticks). Three RNA nucleotides (rU_−1_, Neb_0_ (nebularine) and rC_1_) are displayed. Zinc-coordinating residues H53 (helix α2), C83 and C86 (helix α3) are shown in stick representation (carbons, white; nitrogens, blue; oxygen, red; sulfurs, yellow). (**c**) rA3G-NTD structure (grey ribbon) bound to ssDNA (orange sticks), only dT_0_ has the base while the backbone of dT_1_ and the sugar of dT_0_ is mapped. Surface representation of the nucleotide-binding site of (**d**) A3A, (**e**) TadA (**f**) A3G-NTD. Close-up view of the active site of (**g**) A3A, (**h**) TadA and (**i**) A3G-NTD. The catalytic glutamic acid side chain was modelled in instead of alanine at position 72 in the A3A crystal structure.

The recent structure of ssDNA bound to the inactive pseudo-catalytic domain rA3G-NTD^42^ is not that of a substrate complex and displays a binding mode that is incompatible with catalysis. In contrast to our structure, the single base ordered in that structure is not coordinated within the binding pocket (Supplementary Fig. 5), but rather a sugar is partially buried in the pocket. More specifically, comparing the A3A–ssDNA with the rA3G-NTD–ssDNA structure: H70, W98, S99 and Y130 in A3A (H65, W94, S95 and Y125 in rA3G-NTD) are conserved in the two protein's sequences and interact with ssDNA; however, there are no similarities in their interactions with the ssDNA (Figs 2a and 4; Supplementary Fig. 5). H70 of A3A forms a π-hydrogen bond with dC_0_, while H65 of rA3G-NTD forms a hydrogen bond with C3′-carbonyl group of the ribose of dT_0_. W98 and S99 of A3A use their backbone carbonyl group to hydrogen bond with amino group of the target cytidine (dC_0_), while W94 of rA3G-NTD is stacking with the pyrimidine of dT_0_. Y130 of A3A forms a π–π interaction with dC_0_ and a hydrogen bond with the phosphate between dC_0_ and dT_−1_, while Y125 of rA3G-NTD forms a hydrogen bond with C3′-carbonyl group of the ribose of dT_1_. Many of these interactions preclude the interactions observed in A3A–ssDNA (Figs 1 and 2). Amino acids with more extensive interactions with substrate ssDNA are not conserved in sequence or structure including: H29, which is D, T31, which is V, and the critical N57, which is G (Supplementary Fig. 1). In addition, interactions at −1 and +1 positions are not observed in the rA3G-NTD–ssDNA complex structure as only a single dT_0_ is ordered in the electron density. Critically, the target cytidine (dC_0_) in the A3A is located ready for deamination, while non-substrate dT_0_ in the rA3G-NTD is not located close to the catalytic Zn^2+^. This binding mode corresponds to a much lower affinity of the pseudo-catalytic rA3G-NTD to ssDNA (∼1.6 μM)^42^ confirming non-specific binding, compared to ∼60 nM (ref. 26) we observed for substrate ssDNA binding to A3A. While the rA3G-NTD-dT structure may represent mechanisms by which non-substrate ssDNA binds A3 domains, the A3A–ssDNA structure we present here elucidates the mechanism by which ssDNAs are recognized as substrates by catalytically active A3s.

### Molecular recognition in polynucleotide deaminases

Our crystal structure of the A3A–ssDNA complex and the crystal structure of *Staphylococcus aureus* tRNA adenosine deaminase (TadA) in complex with RNA (2B3J)^60^ are structures of single-stranded polynucleotide deaminases bound to their substrates. Although their substrates are different, as TadA deaminates adenosine at the anti-codon stem-loop of tRNA^Arg2^ and A3A deaminates cytosines in ssDNA, their active sites are similar in that both have a H_A_E_*x*∼30_C_*x*2-4_C zinc-binding motif. We observe the most striking similarity in the phosphate-sugar backbone traces of RNA (TadA) and ssDNA (A3A) (Fig. 4): 5′–3′ directionality is the same, and the polynucleotide is sharply bent with the target nucleotide deep in the active site pocket. Five nucleotides located in the anti-codon stem-loop of tRNA^Arg2^ have adopted C2′-endo ribose conformation that is typical for DNA, explaining how the RNA forms a similar backbone conformation to the ssDNA bound to A3A (Fig. 4a,d,g). This remarkable similarity of the phosphate-sugar backbone, despite different substrates, tRNA for TadA and ssDNA for A3A, implies that the H_A_E_*x*∼30_C_*x*2-4_C type zinc-dependent deaminases have an evolutionary conserved substrate-binding topology as well as catalytic mechanism.

This crystal structure of an ssDNA substrate–enzyme complex reveals how substrate recognition occurs by single-stranded polynucleotide-modifying enzymes, for APOBEC family members and other ssDNA deaminases. This is in contrast with the pseudo-catalytic domain A3G-NTD^42^, which is not a substrate complex and has a single base ordered in the structure that is only partially buried in the binding pocket, displaying a very dissimilar binding mode (Fig. 4c,f,i). The striking similarity of A3A–ssDNA (Fig. 4a,d,g) with the structure of TadA–tRNA complex (Fig. 4b,e,h) implies structural and mechanistic conservation among single-stranded nucleotide-modifying enzymes that have evolved to acquire distinct specificities. These specificities may be leveraged for specific gene editing. APOBEC1 and other cytidine deaminases were recently combined with CRISPR/Cas9 technology in direct ‘base editing' to correct point mutations, without the need for a donor template or double-stranded DNA breaks^61^. By leveraging the directionality, specificity and binding architecture of ssDNA revealed by our A3A–ssDNA complex, base-editing technologies will become even more targeted and specific to expand the scope and effectiveness of genome editing.

## Methods

### Preparation of protein and DNA

The preparation method of A3A(E72A/C171A) protein was described previously^26^ as follows: the protein was expressed in *E. coli* strain BL21 DE3 Star (Stratagene) cells with pCold-GST-A3A(E72A/C171A) vector. Expression was induced with 1 mM isopropyl β-D-1-thiogalactopyranoside at 16 °C for 22 h in lysogeny broth medium containing 100 μg ml^−l^ ampicillin. Cells were pelleted, resuspended in purification buffer (50 mM Tris-HCl (pH 8.0), 300 mM NaCl and 1 mM dithiothreitol) and lysed through sonication. Cellular debris was separated by centrifugation (45,000*g*, 30 min, 4 °C). The protein was purified as a GST-fused protein with glutathione-immobilized resin (Clontech). After digesting with HRV 3C protease, the protein was further purified with a size-exclusion column (GE Healthcare) equilibrated with a buffer (10 mM Tris-HCl (pH 8.0), 200 mM NaCl and 1 mM dithiothreitol). The fraction containing the monomeric form was collected and concentrated for crystallization. The purity and integrity of A3A(E72A/C171A) was confirmed by SDS–polyacrylamide gel electrophoresis. E72A inactivates the enzyme, while C171A (distal the active site) enhances solubility of the expressed protein.

The DNA oligo, d(TTTTTTTTCTTTTTT), was synthesized (Integrated DNA Technologies), and mixed with the purified A3A(E72A/C171A) protein at a molar ratio of 2:1.

### Crystallization and data collection

Crystals of the A3A(E72A/C171A)–DNA complex were grown by hanging-drop vapour-diffusion method over a reservoir of 100 mM MOPS (pH 6.5), 50 mM MgCl_2_, 50 mM CaCl_2_, 23% polyethylene glycol 3,350 and 15% 2-methyl-2,4-pentanediol. Drops were formed by mixing 1 μl of A3A(E72A/C171A)–DNA solution (∼20 mg ml^−l^ of protein concentration) and 1 μl of reservoir solution, with equilibration over the reservoir at 20 °C. Micro-seeding was performed using a cat whisker and larger crystals suitable for X-ray diffraction were obtained. Crystals were flash-frozen directly in the cryogenic stream. Diffraction data were collected using an in-house X-ray source MicroMax-007 HF (Rigaku) with a copper anode at a wavelength of 1.54178 Å and a Saturn 944 HG (Rigaku) detector. The space group of the crystals was I222 with unit cell dimensions of *a*=56.6 Å, *b*=72.7 Å, *c*=115.0 Å (Table 1). The collected intensities were indexed, integrated, corrected for absorption and scaled using HKL2000 (ref. 62).

### Structure determination

The protein structure was solved by molecular replacement phasing using a previously determined apo A3A(E72A/C171A) crystal structure (PDB code 4XXO)^26^ with the program Phaser^63^. Model building of the protein and bound DNA, and refinements were manually performed using the programs Coot^64^ and Phenix^65^,^66^, respectively. A simulated annealing omit map was calculated to confirm the ssDNA positioning (Supplementary Fig. 6). The first nine residues and the side chains of residues R10, H11, H16, K30, N42, V46, K47, Q50, Q58, K60, L62, L63, F66, Y67, D177, E181 and N196 of A3A(E72A/C171A) were not modelled in due to lack of electron density. Residues N42–T44 and L62–G65 were somewhat disordered; the occupancy values were set to 0.5 for residues N42–T44, L62 and G65, and to 0.75 for residues L63 and C64 due to poor electron density. A density proximal to a zinc ion at the active centre was assigned to chloride considering the statistics of zinc ligand^67^, resulting in a good fit without phase-error signals (Supplementary Fig. 7). The identification of the active site zinc is further supported by the highest peak, 9.5*σ*, in an anomalous difference Fourier map at this position. A smaller peak at 5.6*σ* in this map is present at the assigned chloride position. The final model was refined to *R*_(work)_/*R*_(free)_ values of 0.177/0.225 at 2.20 Å resolution (Table 1). The quality of the final model was assessed by Molprobity^68^, which indicated that 96.2% of the residues were in the favoured dihedral angle configuration and there were no Ramachandran outliers.

### Structure analysis

Figures of structure models were generated by Pymol^69^, which was also used to model in the catalytic E72 side chain in Figs 3 and 4. The electrostatic distribution of A3A(E72A/C171A) was calculated and visualized using PDB2PQR server^70^ and Pymol with the APBS plugin, where the cysteine was modelled as thiolate anion (S^−^) and solutes were excluded. Solvent-accessible and buried surface area was calculated with PISA^71^. Local root mean square deviation between apo and DNA-bound forms of A3A(E72A/C171A) was calculated using Molmol^72^. The distance difference matrices between the apo- and DNA-bound forms of A3A(E72A/C171A) were calculated and visualized using a custom-made script in MacOS Xcode (https://developer.apple.com/xcode/).

### Data availability

Atomic coordinates and structural factors for the reported crystal structure have been deposited in the Protein Data Bank http://www.wwpdb.org/ under the accession number 5KEG. The data that support the findings of this study are available from the corresponding author upon request.

## Additional information

**How to cite this article:** Kouno, T. *et al*. Crystal structure of APOBEC3A bound to single-stranded DNA reveals structural basis for cytidine deamination and specificity. *Nat. Commun.* **8,** 15024 doi: 10.1038/ncomms15024 (2017).

**Publisher's note:** Springer Nature remains neutral with regard to jurisdictional claims in published maps and institutional affiliations.

## Supplementary Material

Supplementary Information Supplementary Table and Supplementary Figures

## Figures and Tables

**Table 1:** Data collection and refinement statistics (molecular replacement).

	**A3A/DNA complex**
*Data collection*	
Space group	I222
Cell dimensions	
*a*, *b*, *c* (Å)	56.6, 72.7, 115.0
*α*, *β*, *γ* (°)	90.0, 90.0, 90.0
Resolution (Å)	50.00–2.20 (2.24–2.20)*
*R*_merge_	9.1 (52.8)
*I*/*σI*	27.9 (3.0)
Completeness (%)	98.2 (82.9)
Redundancy	13.1 (8.4)
	
*Refinement*	
Resolution (Å)	50.00–2.20
No. of reflections	11,542
*R*_work_/*R*_free_	0.177/0.225
No. of atoms	
Protein	1,469
Ligand/ion	71/3
Water	100
B-factors	
Protein	35.8
Ligand/ion	50.9/55.1
Water	38.7
r.m.s. deviations	
Bond lengths (Å)	0.011
Bond angles (°)	1.040

^*^Highest-resolution shell is shown in parenthesis.

## References

[b1] Sawyer S. L., Emerman M. & Malik H. S. Ancient adaptive evolution of the primate antiviral DNA-editing enzyme APOBEC3G. PLoS Biol. 2, E275 (2004). 15269786 10.1371/journal.pbio.0020275 PMC479043

[b2] Betts L., Xiang S., Short S. A., Wolfenden R. & Carter C. W. Jr Cytidine deaminase. The 2.3A crystal structure of an enzyme: transition-state analog complex. J. Mol. Biol. 235, 635–656 (1994). 8289286 10.1006/jmbi.1994.1018

[b3] Jarmuz A. . An anthropoid-specific locus of orphan C to U RNA-editing enzymes on chromosome 22. Genomics 79, 285–296 (2002). 11863358 10.1006/geno.2002.6718

[b4] Wedekind J. E., Dance G. S., Sowden M. P. & Smith H. C. Messenger RNA editing in mammals: new members of the APOBEC family seeking roles in the family business. Trends Genet. 19, 207–216 (2003). 12683974 10.1016/S0168-9525(03)00054-4

[b5] Conticello S. G., Thomas C. J., Petersen-Mahrt S. K. & Neuberger M. S. Evolution of the AID/APOBEC family of polynucleotide (deoxy)cytidine deaminases. Mol. Biol. Evol. 22, 367–377 (2005). 15496550 10.1093/molbev/msi026

[b6] LaRue R. S. . Guidelines for naming nonprimate APOBEC3 genes and proteins. J. Virol. 83, 494–497 (2009). 18987154 10.1128/JVI.01976-08 PMC2612408

[b7] Sheehy A. M., Gaddis N. C., Choi J. D. & Malim M. H. Isolation of a human gene that inhibits HIV-1 infection and is suppressed by the viral Vif protein. Nature 418, 646–650 (2002). 12167863 10.1038/nature00939

[b8] Sheehy A. M., Gaddis N. C. & Malim M. H. The antiretroviral enzyme APOBEC3G is degraded by the proteasome in response to HIV-1 Vif. Nat. Med. 9, 1404–1407 (2003). 14528300 10.1038/nm945

[b9] Malim M. H. & Emerman M. HIV-1 accessory proteins--ensuring viral survival in a hostile environment. Cell Host Microbe 3, 388–398 (2008). 18541215 10.1016/j.chom.2008.04.008

[b10] Malim M. H. APOBEC proteins and intrinsic resistance to HIV-1 infection. Philos. Trans. R. Soc. Lond. B Biol. Sci. 364, 675–687 (2009). 19038776 10.1098/rstb.2008.0185 PMC2660912

[b11] Koning F. A., Goujon C., Bauby H. & Malim M. H. Target cell-mediated editing of HIV-1 cDNA by APOBEC3 proteins in human macrophages. J. Virol. 85, 13448–13452 (2011). 21957290 10.1128/JVI.00775-11 PMC3233168

[b12] Berger G. . APOBEC3A is a specific inhibitor of the early phases of HIV-1 infection in myeloid cells. PLoS Pathog. 7, e1002221 (2011). 21966267 10.1371/journal.ppat.1002221 PMC3178557

[b13] Harris R. S., Hultquist J. F. & Evans D. T. The restriction factors of human immunodeficiency virus. J. Biol. Chem. 287, 40875–40883 (2012). 23043100 10.1074/jbc.R112.416925 PMC3510791

[b14] Chan K. . An APOBEC3A hypermutation signature is distinguishable from the signature of background mutagenesis by APOBEC3B in human cancers. Nat. Genet. 47, 1067–1072 (2015). 26258849 10.1038/ng.3378 PMC4594173

[b15] Hoopes J. I. . APOBEC3A and APOBEC3B preferentially deaminate the lagging strand template during DNA replication. Cell Rep. 14, 1273–1282 (2016). 26832400 10.1016/j.celrep.2016.01.021 PMC4758883

[b16] Kazanov M. D. . APOBEC-induced cancer mutations are uniquely enriched in early-replicating, gene-dense, and active chromatin regions. Cell Rep 13, 1103–1109 (2015). 26527001 10.1016/j.celrep.2015.09.077 PMC4644490

[b17] Roberts S. A. . An APOBEC cytidine deaminase mutagenesis pattern is widespread in human cancers. Nat. Genet. 45, 970–976 (2013). 23852170 10.1038/ng.2702 PMC3789062

[b18] Burns M. B. . APOBEC3B is an enzymatic source of mutation in breast cancer. Nature 494, 366–370 (2013). 23389445 10.1038/nature11881 PMC3907282

[b19] Burns M. B., Temiz N. A. & Harris R. S. Evidence for APOBEC3B mutagenesis in multiple human cancers. Nat. Genet. 45, 977–983 (2013). 23852168 10.1038/ng.2701 PMC3902892

[b20] Sakofsky C. J. . Break-induced replication is a source of mutation clusters underlying kataegis. Cell Rep. 7, 1640–1648 (2014). 24882007 10.1016/j.celrep.2014.04.053 PMC4274036

[b21] Chen K. M. . Structure of the DNA deaminase domain of the HIV-1 restriction factor APOBEC3G. Nature 452, 116–119 (2008). 18288108 10.1038/nature06638

[b22] Harjes E. . An extended structure of the APOBEC3G catalytic domain suggests a unique holoenzyme model. J. Mol. Biol. 389, 819–832 (2009). 19389408 10.1016/j.jmb.2009.04.031 PMC2700007

[b23] Shandilya S. M. . Crystal structure of the APOBEC3G catalytic domain reveals potential oligomerization interfaces. Structure 18, 28–38 (2010). 20152150 10.1016/j.str.2009.10.016 PMC2913127

[b24] Li M. . First-in-class small molecule inhibitors of the single-strand DNA cytosine deaminase APOBEC3G. ACS Chem. Biol. 7, 506–517 (2012). 22181350 10.1021/cb200440y PMC3306499

[b25] Bohn M. F. . Crystal structure of the DNA cytosine deaminase APOBEC3F: the catalytically active and HIV-1 Vif-binding domain. Structure 21, 1042–1050 (2013). 23685212 10.1016/j.str.2013.04.010 PMC3805256

[b26] Bohn M. F. . The ssDNA mutator APOBEC3A is regulated by cooperative dimerization. Structure 23, 903–911 (2015). 25914058 10.1016/j.str.2015.03.016 PMC4874493

[b27] Kouno T. . Structure of the Vif-binding domain of the antiviral enzyme APOBEC3G. Nat. Struct. Mol. Biol. 22, 485–491 (2015). 25984970 10.1038/nsmb.3033 PMC4456288

[b28] Chelico L., Pham P., Calabrese P. & Goodman M. F. APOBEC3G DNA deaminase acts processively 3′ --> 5′ on single-stranded DNA. Nat. Struct. Mol. Biol. 13, 392–399 (2006). 16622407 10.1038/nsmb1086

[b29] Holden L. G. . Crystal structure of the anti-viral APOBEC3G catalytic domain and functional implications. Nature 456, 121–124 (2008). 18849968 10.1038/nature07357 PMC2714533

[b30] Chelico L., Sacho E. J., Erie D. A. & Goodman M. F. A model for oligomeric regulation of APOBEC3G cytosine deaminase-dependent restriction of HIV. J. Biol. Chem. 283, 13780–13791 (2008). 18362149 10.1074/jbc.M801004200 PMC2376223

[b31] Furukawa A. . Structure, interaction and real-time monitoring of the enzymatic reaction of wild-type APOBEC3G. EMBO J. 28, 440–451 (2009). 19153609 10.1038/emboj.2008.290 PMC2646150

[b32] Chelico L., Prochnow C., Erie D. A., Chen X. S. & Goodman M. F. A structural model for deoxycytidine deamination mechanisms of the HIV-1 inactivation enzyme APOBEC3G. J. Biol. Chem. 285, 16195–16205 (2010). 20212048 10.1074/jbc.M110.107987 PMC2871487

[b33] Kitamura S. . The APOBEC3C crystal structure and the interface for HIV-1 Vif binding. Nat. Struct. Mol. Biol. 19, 1005–1010 (2012). 23001005 10.1038/nsmb.2378

[b34] Siu K. K., Sultana A., Azimi F. C. & Lee J. E. Structural determinants of HIV-1 Vif susceptibility and DNA binding in APOBEC3F. Nat. Commun. 4, 2593 (2013). 24185281 10.1038/ncomms3593 PMC4956467

[b35] Byeon I. J. . NMR structure of human restriction factor APOBEC3A reveals substrate binding and enzyme specificity. Nat. Commun. 4, 1890 (2013). 23695684 10.1038/ncomms2883 PMC3674325

[b36] Mitra M. . Structural determinants of human APOBEC3A enzymatic and nucleic acid binding properties. Nucleic Acids Res. 42, 1095–1110 (2014). 24163103 10.1093/nar/gkt945 PMC3902935

[b37] Lu X. . Crystal structure of DNA cytidine deaminase ABOBEC3G catalytic deamination domain suggests a binding mode of full-length enzyme to single-stranded DNA. J. Biol. Chem. 290, 4010–4021 (2015). 25542899 10.1074/jbc.M114.624262 PMC4326812

[b38] Shi K., Carpenter M. A., Kurahashi K., Harris R. S. & Aihara H. Crystal structure of the DNA deaminase APOBEC3B catalytic domain. J. Biol. Chem. 290, 28120–28130 (2015). 26416889 10.1074/jbc.M115.679951 PMC4653671

[b39] Nakashima M. . Structural insights into HIV-1 Vif-APOBEC3F interaction. J. Virol. 90, 1034–1047 (2015). 26537685 10.1128/JVI.02369-15 PMC4702671

[b40] Shaban N. M., Shi K., Li M., Aihara H. & Harris R. S. 1.92 Angstrom zinc-free APOBEC3F catalytic domain crystal structure. J. Mol. Biol. 428, 2307–2316 (2016). 27139641 10.1016/j.jmb.2016.04.026 PMC5142242

[b41] Byeon I. J. . Nuclear magnetic resonance structure of the APOBEC3B catalytic domain: structural basis for substrate binding and DNA deaminase activity. Biochemistry 55, 2944–2959 (2016). 27163633 10.1021/acs.biochem.6b00382 PMC4943463

[b42] Xiao X., Li S. X., Yang H. & Chen X. S. Crystal structures of APOBEC3G N-domain alone and its complex with DNA. Nat. Commun. 7, 12193 (2016). 27480941 10.1038/ncomms12193 PMC4974639

[b43] Shandilya S. M., Bohn M. F. & Schiffer C. A. A computational analysis of the structural determinants of APOBEC3's catalytic activity and vulnerability to HIV-1 Vif. Virology 471–473, 105–116 (2014). 10.1016/j.virol.2014.09.023 PMC4857191 25461536

[b44] Carlow D. C., Short S. A. & Wolfenden R. Complementary truncations of a hydrogen bond to ribose involved in transition-state stabilization by cytidine deaminase. Biochemistry 37, 1199–1203 (1998). 9477944 10.1021/bi971731n

[b45] Snider M. J., Reinhardt L., Wolfenden R. & Cleland W. W. 15N kinetic isotope effects on uncatalyzed and enzymatic deamination of cytidine. Biochemistry 41, 415–421 (2002). 11772041 10.1021/bi011410i

[b46] Chen H. . APOBEC3A is a potent inhibitor of adeno-associated virus and retrotransposons. Curr. Biol. 16, 480–485 (2006). 16527742 10.1016/j.cub.2006.01.031

[b47] Chiu Y. L. & Greene W. C. The APOBEC3 cytidine deaminases: an innate defensive network opposing exogenous retroviruses and endogenous retroelements. Annu. Rev. Immunol. 26, 317–353 (2008). 18304004 10.1146/annurev.immunol.26.021607.090350

[b48] Goila-Gaur R. & Strebel K. HIV-1 Vif, APOBEC, and intrinsic immunity. Retrovirology 5, 51 (2008). 18577210 10.1186/1742-4690-5-51 PMC2443170

[b49] Furukawa A. . Quantitative analysis of location- and sequence-dependent deamination by APOBEC3G using real-time NMR spectroscopy. Angew. Chem. Int. Ed. 53, 2349–2352 (2014). 10.1002/anie.201309940 24478136

[b50] Carpenter M. A. . Methylcytosine and normal cytosine deamination by the foreign DNA restriction enzyme APOBEC3A. J. Biol. Chem. 287, 34801–34808 (2012). 22896697 10.1074/jbc.M112.385161 PMC3464582

[b51] Pham P., Landolph A., Mendez C., Li N. & Goodman M. F. A biochemical analysis linking APOBEC3A to disparate HIV-1 restriction and skin cancer. J. Biol. Chem. 288, 29294–29304 (2013). 23979356 10.1074/jbc.M113.504175 PMC3795231

[b52] Bulliard Y. . Structure-function analyses point to a polynucleotide-accommodating groove essential for APOBEC3A restriction activities. J. Virol. 85, 1765–1776 (2011). 21123384 10.1128/JVI.01651-10 PMC3028873

[b53] Sharma S., Patnaik S. K., Kemer Z. & Baysal B. E. Transient overexpression of exogenous APOBEC3A causes C-to-U RNA editing of thousands of genes. RNA Biol http://dx.doi.org/10.1080/15476286.2016.1184387 (2016). 10.1080/15476286.2016.1184387 PMC5449087 27149507

[b54] Sharma S. . APOBEC3A cytidine deaminase induces RNA editing in monocytes and macrophages. Nat. Commun. 6, 6881 (2015). 25898173 10.1038/ncomms7881 PMC4411297

[b55] Teh A. H. . The 1.48A resolution crystal structure of the homotetrameric cytidine deaminase from mouse. Biochemistry 45, 7825–7833 (2006). 16784234 10.1021/bi060345f

[b56] Marx A. & Alian A. The first crystal structure of a dTTP-bound deoxycytidylate deaminase validates and details the allosteric-inhibitor binding site. J. Biol. Chem. 290, 682–690 (2015). 25404739 10.1074/jbc.M114.617720 PMC4281768

[b57] Chung S. J., Fromme J. C. & Verdine G. L. Structure of human cytidine deaminase bound to a potent inhibitor. J. Med. Chem. 48, 658–660 (2005). 15689149 10.1021/jm0496279

[b58] Marx A., Galilee M. & Alian A. Zinc enhancement of cytidine deaminase activity highlights a potential allosteric role of loop-3 in regulating APOBEC3 enzymes. Sci. Rep. 5, 18191 (2015). 26678087 10.1038/srep18191 PMC4683357

[b59] Harjes S. . Impact of H216 on the DNA binding and catalytic activities of the HIV restriction factor APOBEC3G. J. Virol. 87, 7008–7014 (2013). 23596292 10.1128/JVI.03173-12 PMC3676121

[b60] Losey H. C., Ruthenburg A. J. & Verdine G. L. Crystal structure of *Staphylococcus aureus* tRNA adenosine deaminase TadA in complex with RNA. Nat. Struct. Mol. Biol. 13, 153–159 (2006). 16415880 10.1038/nsmb1047

[b61] Komor A. C., Kim Y. B., Packer M. S., Zuris J. A. & Liu D. R. Programmable editing of a target base in genomic DNA without double-stranded DNA cleavage. Nature 533, 420–424 (2016). 27096365 10.1038/nature17946 PMC4873371

[b62] Otwinowski Z. & Minor W. Processing of X-ray diffraction data collected in oscillation mode. Methods Enzymol. 276, 307–326 (1997). 10.1016/S0076-6879(97)76066-X 27754618

[b63] Bunkoczi G. . Phaser.MRage: automated molecular replacement. Acta Crystallogr. D Biol. Crystallogr. 69, 2276–2286 (2013). 24189240 10.1107/S0907444913022750 PMC3817702

[b64] Emsley P. & Cowtan K. Coot: model-building tools for molecular graphics. Acta Crystallogr. D Biol. Crystallogr. 60, 2126–2132 (2004). 15572765 10.1107/S0907444904019158

[b65] Adams P. D. . The Phenix software for automated determination of macromolecular structures. Methods 55, 94–106 (2011). 21821126 10.1016/j.ymeth.2011.07.005 PMC3193589

[b66] Echols N. . Graphical tools for macromolecular crystallography in PHENIX. J. Appl. Crystallogr. 45, 581–586 (2012). 22675231 10.1107/S0021889812017293 PMC3359726

[b67] Laitaoja M., Valjakka J. & Janis J. Zinc coordination spheres in protein structures. Inorg. Chem. 52, 10983–10991 (2013). 24059258 10.1021/ic401072d

[b68] Chen V. B. . MolProbity: all-atom structure validation for macromolecular crystallography. Acta Crystallogr. D Biol. Crystallogr. 66, 12–21 (2010). 20057044 10.1107/S0907444909042073 PMC2803126

[b69] Schrödinger L. L. C. The PyMOL Molecular Graphics System, Version 1.8 (2015).

[b70] Dolinsky T. J., Nielsen J. E., McCammon J. A. & Baker N. A. PDB2PQR: an automated pipeline for the setup of Poisson-Boltzmann electrostatics calculations. Nucleic Acids Res. 32, W665–W667 (2004). 15215472 10.1093/nar/gkh381 PMC441519

[b71] Krissinel E. & Henrick K. Inference of macromolecular assemblies from crystalline state. J. Mol. Biol. 372, 774–797 (2007). 17681537 10.1016/j.jmb.2007.05.022

[b72] Koradi R., Billeter M. & Wuthrich K. MOLMOL: a program for display and analysis of macromolecular structures. J. Mol. Graph. 14, 51–55 (1996). 8744573 10.1016/0263-7855(96)00009-4

