# Supplementary material for: Crystal structure of APOBEC3A bound to single-stranded DNA reveals structural basis for cytidine deamination and specificity
Source: Nat Commun. 2017 Apr 28;8:15024. doi: 10.1038/ncomms15024 (PMC5414352; doi:10.1038/ncomms15024)
Supplement: Supplementary Information — Supplementary Table and Supplementary Figures [file ncomms15024-s1.pdf]

Residue (A3A sequence)	**Catalytic**	**Inactive pseudo-catalytic**
Included Domains	A3A, A3B-CTD, A3C, A3D-CTD, A3G-CTD, A3F-CTD	A3B-NTD, A3D-NTD, A3G-NTD, (rA3G-NTD) A3F-NTD
28	**Arg**	**Arg** (ex A3G-NTD Leu)
29	His / Asn / Arg	Asn / Asp / Ser
31	Ser / Thr	Val / Thr
57	**Asn**	**Gly**
70	**His (Zn)**	**His (Zn)**
71	**Ala**	Ala / Pro
72	**Glu (catalytic)**	**Glu (Zn)**
98	**Trp**	**Trp**
99	**Ser**	Ser / Thr / Asn
101	**Cys (Zn)**	**Cys (Zn)**
106	**Cys (Zn)**	**Cys (Zn)**
130	**Tyr**	**Tyr**
131	Asp / Tyr	**Tyr**
132	Asp / Tyr / Phe	Phe / Tyr
189	Arg / Lys	Met / Thr

*APOBEC3H not included

Supplementary Table 1. Conservation of DNA coordinating residues between human A3 domains (catalytic and inactive pseudo-catalytic)

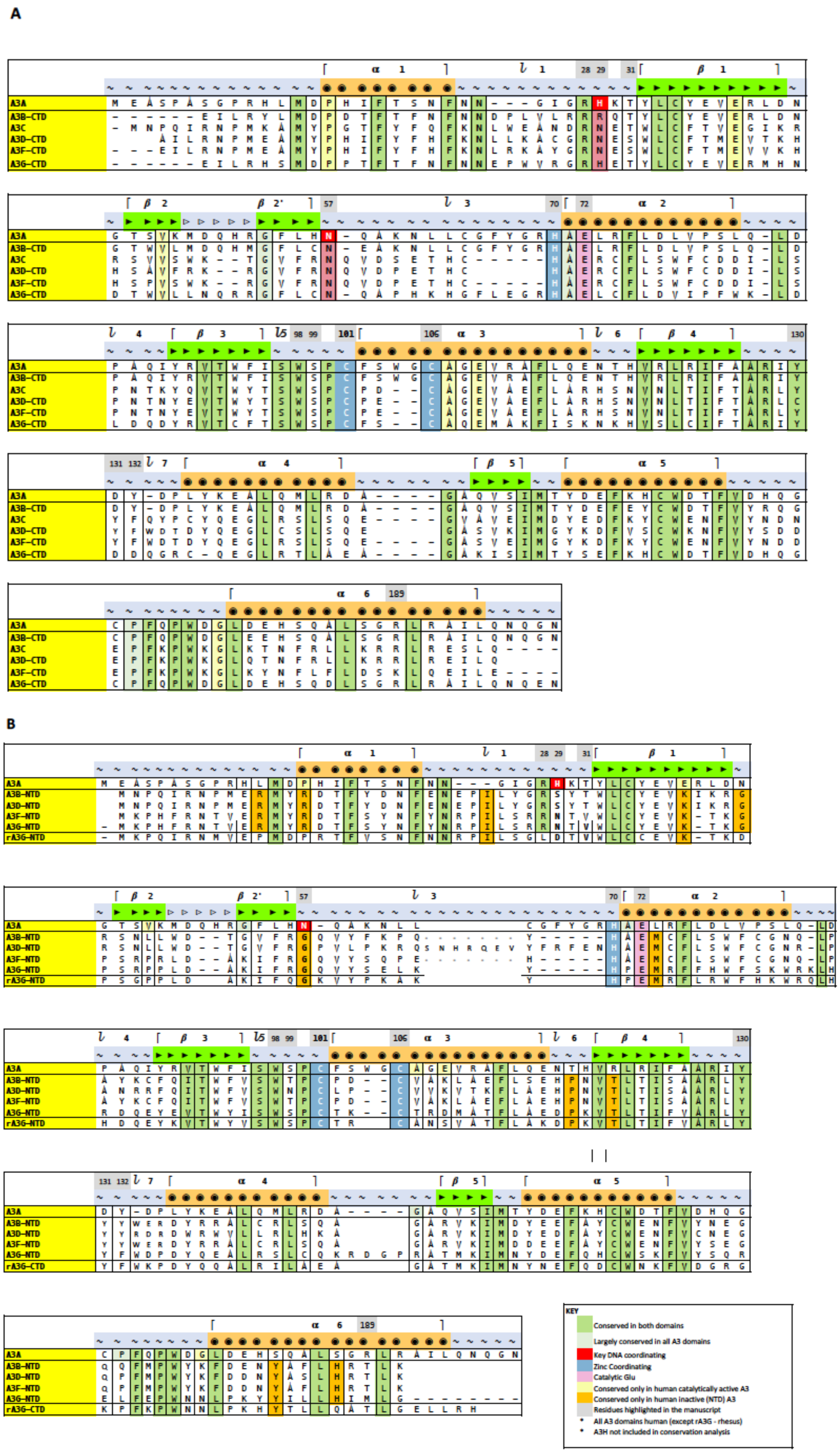

Supplementary Fig. 1. Sequence alignment of A3A A) with sequences of catalytically active A3 domains whose crystal structures have been determined, B) with sequences of inactive pseudo-catalytic domains whose structures have been solved.

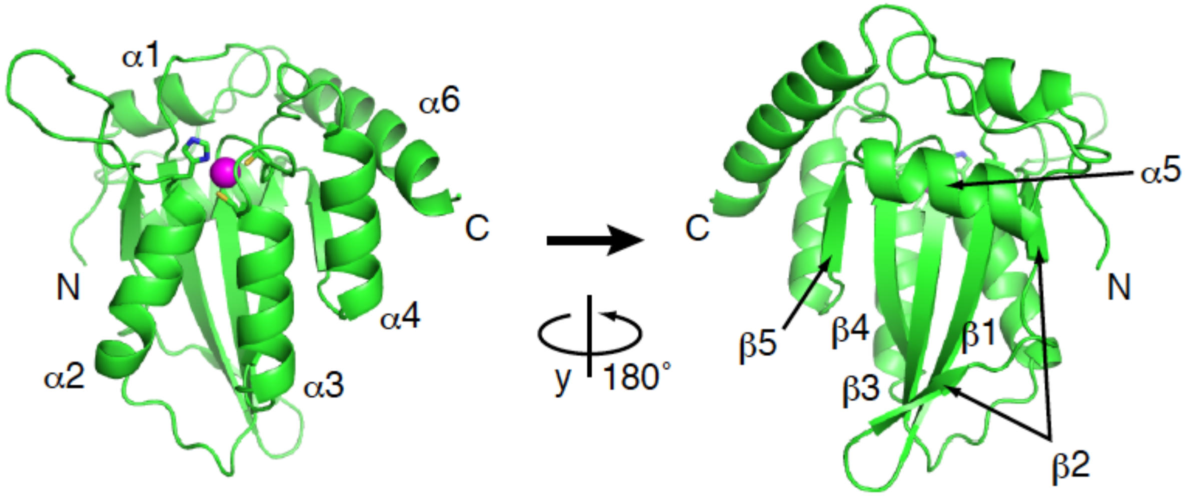

Supplementary Fig. 2. Secondary structure elements of A3A. A3A structure is shown as a ribbon diagram in green. A zinc ion at the active site is depicted as a magenta-colored sphere. The zinc-coordinating residues H70, C101 and C106 are in stick representation (carbons, green; nitrogens, blue; oxygens, red; sulfurs, yellow). A3A structure is comprised of helix α1 (residues 15–22), strand β1 (32–41), strand β2 (44–47 and 53–56, with a break or "kink" in the strand), helix α2 (71–82), strand β3 (89–96), helix α3 (106–116), strand β4 (120–126), helix α4 (136–146), strand β5 (149–152), helix α5 (155–165) and helix α6 (179–195).

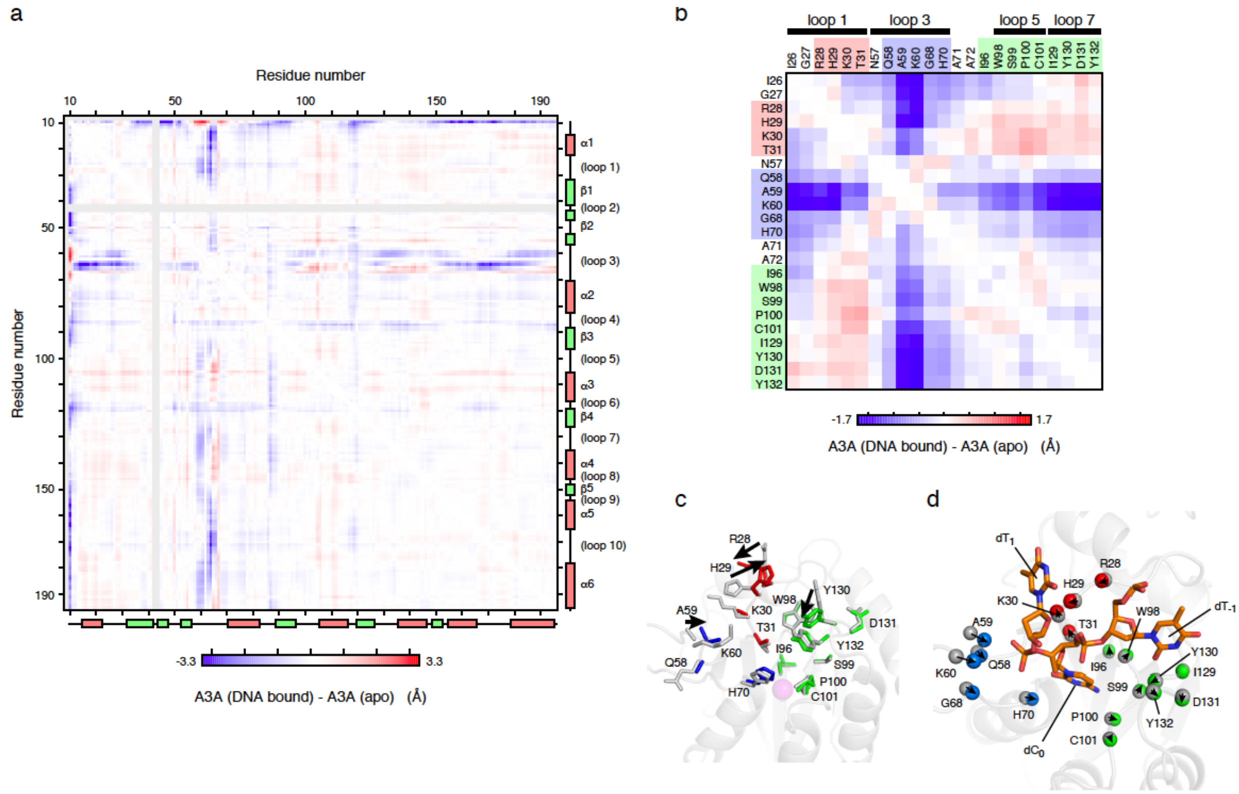

Supplementary Fig. 3. (a) Distance difference matrix between apo (PDB code 4XXO) [26] and DNA-bound form of A3A. All possible inter-Cα distances were calculated within the apo and DNA-bound form of A3A. Each distance in the apo form was subtracted from the corresponding distance in the DNA-bound form, and the resultant distance difference matrix is displayed as a contour plot (blue and red for negative and positive values, respectively, with a scale of -3.3 to +3.3 Å). The secondary structure elements of the DNA-bound A3A are indicated along the matrix; red- and green-colored rectangles depict α-helices and β-strands, respectively. (b) The distance difference matrix of selected A3A residues forming the interface with the bound DNA, showing the changes between apo and DNA-bound structures. The residues were grouped into three categories and indicated by red, green and blue; red- and green-colored residues have a relatively longer distance to each other when the substrate DNA binds to A3A whereas blue-colored residues have a shorter distance to both red- and green-colored residues. The location of selected residues in the three groups on the A3A structure, (c) side chains displayed in stick representation with arrows indicating side chain conformational changes, (d) spheres indicating the position of Cα atoms (apo gray, bound colored according to the three groups as in panel a). The Cα position changes upon the DNA binding are depicted by arrows. DNA molecule bound to A3A is in stick representation (carbons and phosphates, orange; nitrogens, blue; oxygens, red) and three nucleotides (dT_{-1}, dC_0 and dT_1) are shown.

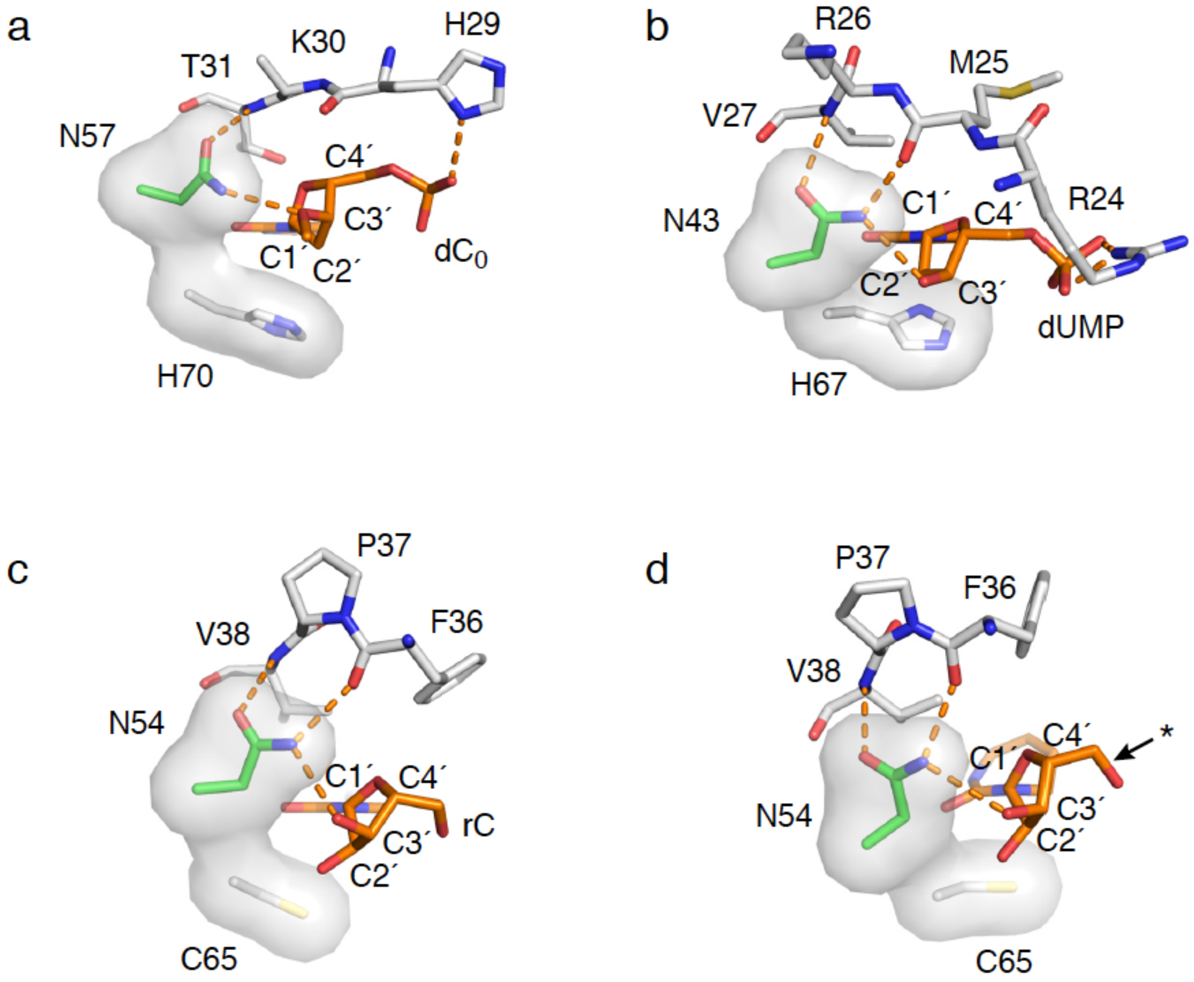

Supplementary Fig. 4. Close-up views of the conserved asparagine and sugar arrangement. The selected amino acid residues in (a) A3A in complex with ssDNA (this study; only dC0 is shown) (b) DNA cytidine deaminase from bacteriophage S-TIM5 (PDB code 4P9C)[56] in complex with deoxyuridine monophosphate (dUMP) (c) RNA cytidine deaminase from mouse (PDB code 2FR6)[55] in complex with cytidine (rC) and (d) RNA cytidine deaminase from human (PDB code 1MQ0)[57] in complex with deaminase inhibitor, 1-β- ribofuranosyl-1,3-diazapinone (indicated by asterisk) are shown as a stick model (carbons for the conserved asparagine residue, green; carbons for others, white). The substrates are colored orange for carbons and phosphates. The conserved asparagine and neighboring histidine/cysteine are drawn with van der Waals surface.

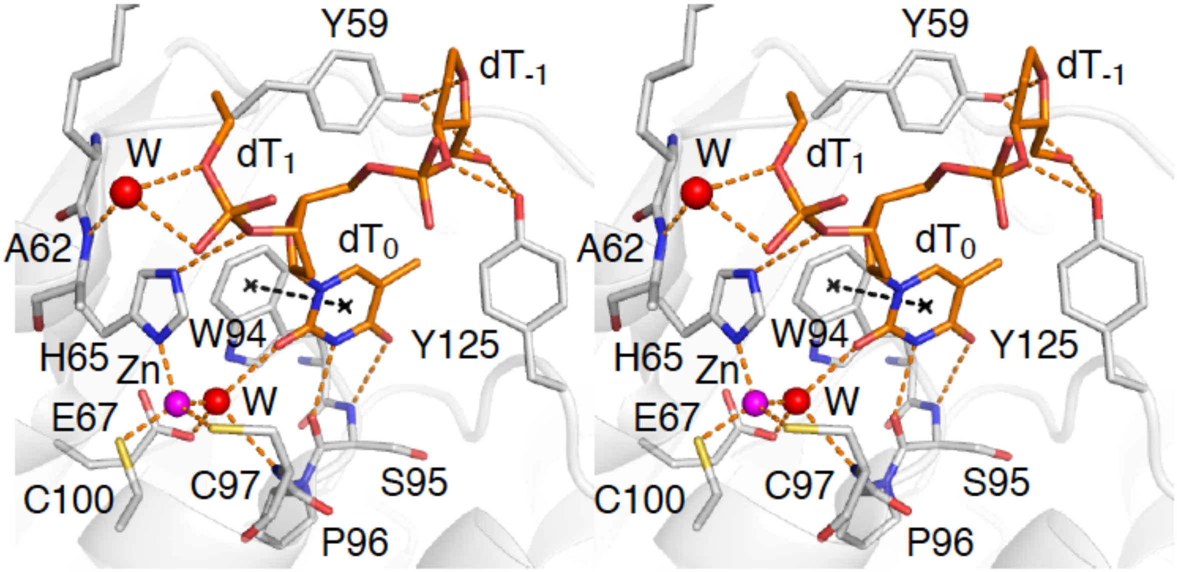

Supplementary Fig. 5. Stereo-view of the atomic interactions between rA3G-NTD and ssDNA (5K83). rA3G-NTD (gray ribbon) bound to ssDNA (orange sticks: dT_0 base, the backbone of dT_1 and the sugar of dT_0), magenta and red spheres are water and Zn respectively.

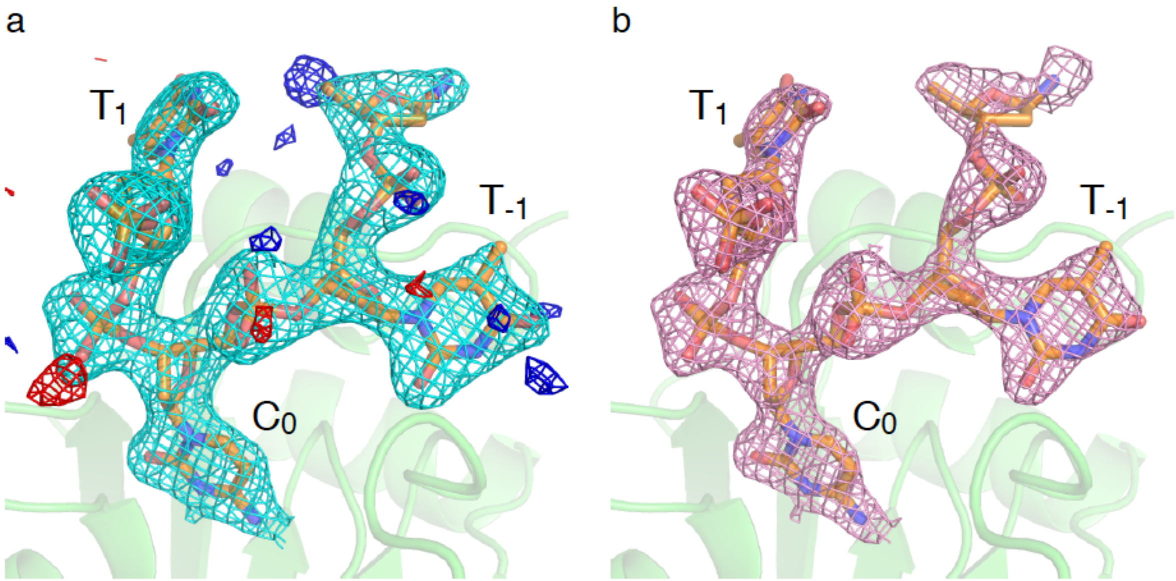

Supplementary Fig. 6. **(a)** 2*F*o-*F*c and *F*o-*F*c maps of the bound DNA. A3A and DNA structure are represented as cartoon and stick model, respectively. The 2*F*o-*F*c map is indicated with a cyan-colored mesh (1.0σ), the *F*c-*F*o map is depicted *blue* (3.0σ) and *red* (-3.0σ). **(b)** The simulated-annealing composite omit map of the bound DNA. The 2*mF*o-*DF*c map (*pink)* is contoured at 1.0σ.

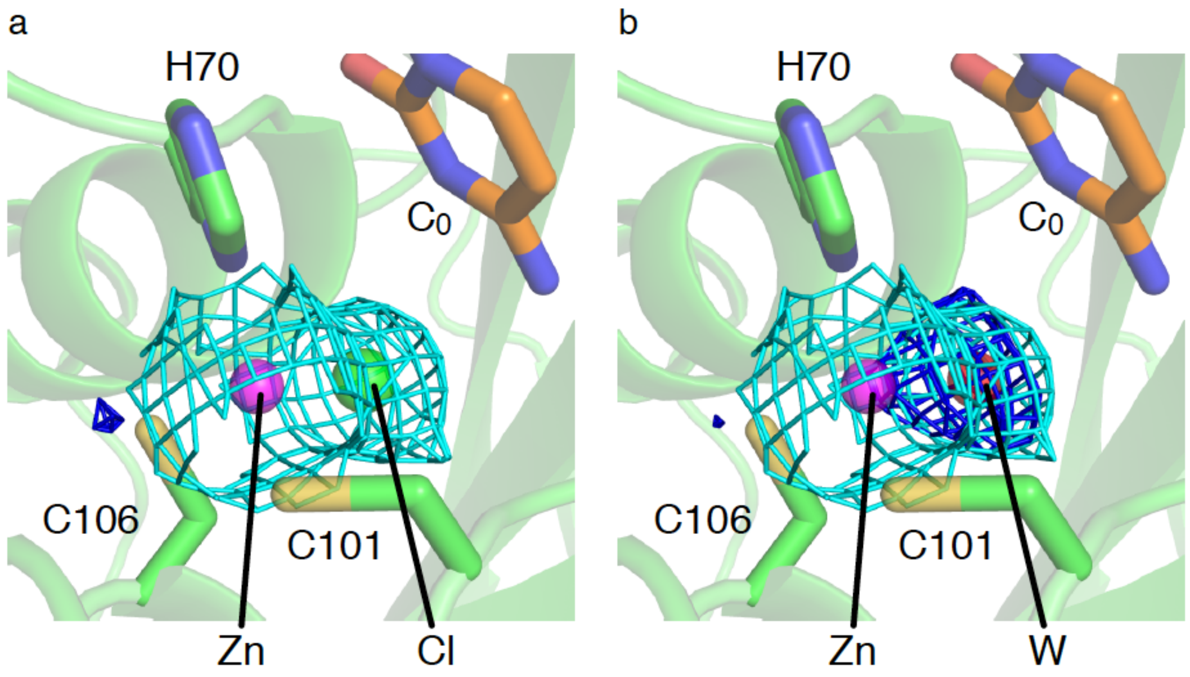

Supplementary Fig. 7 Modeling in the electron density next to the active site zinc (Zn) with a (a) chloride ion (Cl) versus (b) water molecule (W). The 2*F*o-*F*c map is indicated with a cyan-colored mesh (1.0σ), the *F*c-*F*o map is depicted by meshes colored in *blue* (3.0σ) and *red* (-3.0σ).
